# Systematic evaluation of petroleum sulfonate: polarity separation and the relationship between its structure and oil recovery properties

**DOI:** 10.1039/c8ra06739b

**Published:** 2018-10-02

**Authors:** Yawei Duan, Youyi Zhu, Jian Fan, Wenjun Li, Xintong Liu, Hongda Li

**Affiliations:** Beijing Key Laboratory for Science and Application of Functional Molecular and Crystalline Materials, University of Science and Technology Beijing Beijing 100083 China wjli_ustb@163.com; Research Institute of Petroleum Exploration and Development, China National Petroleum Corporation Beijing 100083 China

## Abstract

Petroleum sulfonate is one of the most important surfactants in the tertiary oil recovery process. However, its complex composition significantly impedes its evaluation, and the relationship between its structure and oil recovery properties is still unclear. In this study, the actives of petroleum sulfonate are subdivided into seven components, a–g, with different polarities *via* column chromatography. The structural information of each component is fully characterized. Moreover, the relationship between the oil recovery properties and the structure of the separated components is systematically studied. The results reveal the average relative molecular mass in the range of 560–626, average alkyl side chain containing 36–40 carbon atoms and alkyl chain containing an average of 6 branched chains is the ideal structure for enhancing oil recovery properties. Furthermore, this study provides a reliable evaluation method and reveals the relationship between the structure and oil recovery properties of petroleum sulfonate.

## Abstract 2

Petroleum sulfonate actives are efficiently separated and fully characterized, and the relationship between their structure and oil recovery properties is revealed.

## Graphical abstract

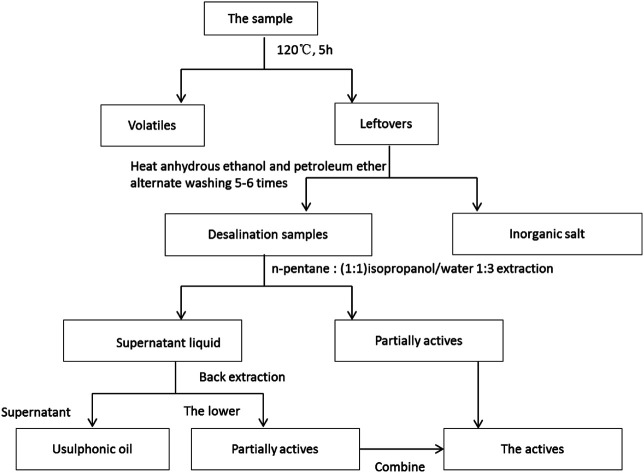

## 1 Introduction

Petroleum sulfonate, as an anionic surfactant, has attracted increasing attention in chemical flooding due to its high capacity and low cost.^1,2^ Petroleum sulfonate products are complex mixtures consisting of volatiles, inorganic salts, unsulfonated oil and different molecular structure actives.^3–5^ To date, many researchers have mainly focused on the determination of overall actives.^6,7^ However, the diversity of the active structures of petroleum sulfonate results in a great difference in oil recovery performance; thus, current research to determine the content of overall actives cannot accurately evaluate petroleum sulfonate.^8–10^ Hence, it is of great significance to further separate the actives and investigate the structure and properties of the separated samples.

Previous research on alkylbenzene sulfonate surfactant showed that different components have different polarities and specific low polarity sulfonate components show significant ultra-low interfacial tension. Therefore, the petroleum sulfonate components with different polarities may also show different oil recovery properties, and polarity separation may be a considerable method for the further separation of petroleum sulfonate actives.^11^ The separated samples with different structures will show different dynamic interfacial tension; however, the relationship between their structure and properties is still unclear. Hence, insight into separated samples may be of great significance in the area of oil recovery.^12,13^ Significantly, this study aims to provide guidance for the evaluation and industrial production of petroleum sulfonate.

In this study, the actives from a petroleum sulfonate sample are purified and subdivided into seven components. DIT is the significant and reliable indicator for evaluating surfactants for chemical flooding, meanwhile ES is also used to further evaluate the emulsifying properties of surfactants. Accordingly, the effective components of petroleum sulfonate are identified by measuring the DIT and ES of each component. The structures of the seven components such as their types of functional groups, aromaticity, and mass distribution are fully characterized and represented in detail. Additionally, relationship between the structure and interfacial chemical properties of the petroleum sulfonate components are reasonably deduced. This study provides an efficient evaluation method and guide for the industrial production of petroleum sulfonate.

## 2 Experimental

### 2.1 Separation of petroleum sulfonate

Industrial products of petroleum sulfonate contain volatile components, inorganic salts, unsulfonated oil and other impurities. In this experiment, the actives of petroleum sulfonate were purified using the extraction method.^14–17^ Petroleum sulfonate samples were heated to 120 °C and maintained for several hours to remove the volatiles. After cooling to room temperature, the non-volatile leftovers were alternately washed with hot ethanol and petroleum ether until the color turned white, and the residual solid was the inorganic salt. Next, the obtained washing solution was dried using a rotary evaporator.

Afterwards, the obtained desalination samples were dissolved in a mixed solvent (50% isopropanol/water, volume ratio) and repeatedly extracted with *n*-pentane until the supernatant liquid turned yellowish, and then the lower liquid was collected. Then, back extraction of the supernatant liquid was performed. Finally, the actives were obtained. The petroleum sulfonate samples used in the experiment were obtained from the Daqing Oilfield in China. All chemicals were used with no further purification.

### 2.2 Polarity separation of petroleum sulfonate actives

The inner diameter and length of the chromatography column were 45 mm and 300 mm, respectively. The filling height of the column was 150 mm. Before filling the column, the silica gel was activated at 120 °C for 6 h.^18^

To systematically evaluate the different polarity components of petroleum sulfonate, firstly, the samples were divided into two parts: 60% low and 40% high polarity components *via* column chromatography using petroleum ether as the eluant. Next the low and high polarity components were subdivided. Based on the polarity sequence and the polarity properties of petroleum sulfonate,^19^ the elution order was determined as follows.

Separation of low polarity components: methylbenzene → *n*-butanol → acetic acid → deionized water. The successively eluted components were marked as a, b, c and d, respectively.

Separation of high polarity components: ethyl acetate → isopropanol → deionized water. The successively eluted components were marked as e, f and g, respectively.

### 2.3 Determination of oil–water interfacial tension

In this experiment, the dynamic oil–water interfacial tension was determined using a spinning drop surface/interface tensiometer (TX-500C, CNG, USA) at 45 °C and 5000 rpm. The surfactant flooding system (0.3% g L^−1^) configured with mineralized water was used as the water phase. The oil phase was crude oil from the Daqing Oilfield in China. The morphology of the oil drop was recorded at different times. Then, the oil–water interfacial tension of each component was measured. Thus, the main effective components in petroleum sulfonate were determined. Furthermore, the relationship between structure and interfacial tension was established by characterizing the structure of the main effective components. The oil–water interfacial tension was calculated from eqn (1):^20^1
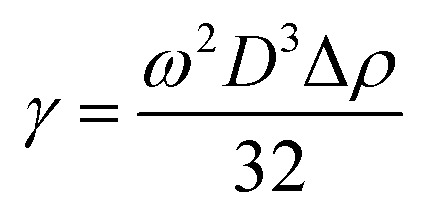
*ω* – angular velocity; *D* – drop diameter; and Δ*ρ* – difference in density between the flooding system and crude oil (Δ*ρ* is 0.15 g mL^−1^).

### 2.4 Determination of emulsification stability (ES)

The emulsifier solution (0.3%) was configured with mineralized water and emulsifier. The Daqing crude oil and emulsifier solution were mixed with a mass ratio of 1 : 1 at 90 °C for 30 min. Then, the mixed solution was stirred with a high speed dispersive emulsifier for 3 min at 11 000 rpm. Then the solution was poured into a measuring cylinder and left to stand for thermal insulation. The volume of the lower water phase (*V*_wi_) at different times was recorded, and the water separating rate (*S*_wi_) at different times calculated using eqn (2-1):2-1
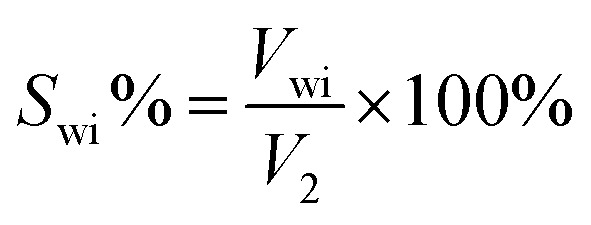
*V*_wi_ – volume of the lower water phase at different times, mL; and*V*_2_ – initial volume of emulsifier solution, mL.

The emulsification stability *S*_te_ was calculated using eqn (2-2):

2-2 *S*_te_ = 1 − *S*_w1_

*S*_w1_ – water separating rate of the emulsion after thermal insulation for 1 h, %; and *S*_te_ – emulsification stability, %.

## 3 Results and discussion

### 3.1 Separation of petroleum sulfonate

Table 1 shows that the recovery rate of the total product is about 98%. Additionally, the recovery rate of actives is 29.04%, which indicates that the crude separation has high credibility.^21^

**Table 1:** Composition of petroleum sulfonates

Component	Mass fraction/%	Recovery rate/%
Volatile	28.27	97.98
Inorganic salt	12.89	
Unsulfated oil	27.78	
Actives	29.04	

**Fig. 1:**
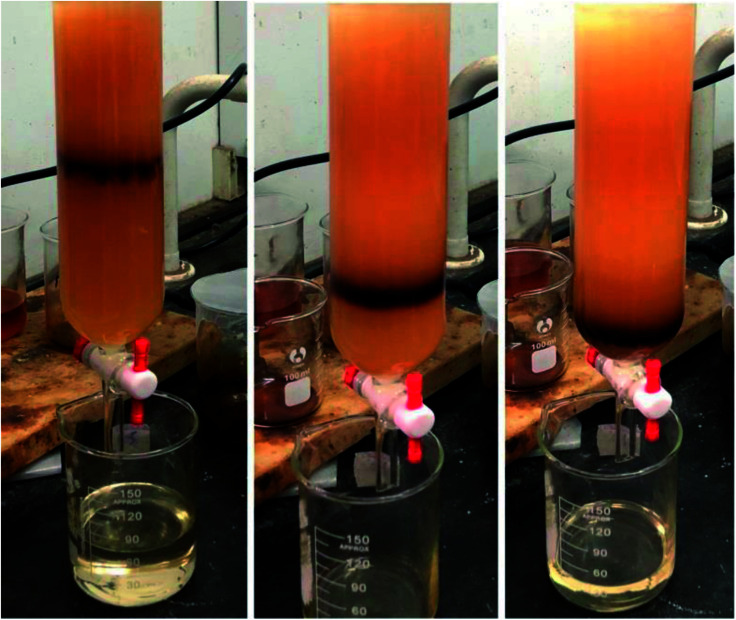
Separation process *via* column chromatography.

The actives were divided into different polarity components *via* column chromatography. As shown in Fig. 1, the eluent ribbon is clear and smooth. The low and high polarity components of petroleum sulfonate were separated using petroleum ether as the eluent, and their mass fraction was about 60% and 40%, respectively. Table 2 shows that the recovery rate of component b is higher than that of the other low polarity components.

**Table 2:** Column chromatography results for the low polarity components

Component number	Eluent	Mass fraction/%
a	Methylbenzene	12.42
b	*n*-Butanol	33.56
c	Acetic acid	3.68
d	Deionized water	6.25

### 3.2 Interfacial tension behavior

**Fig. 2:**
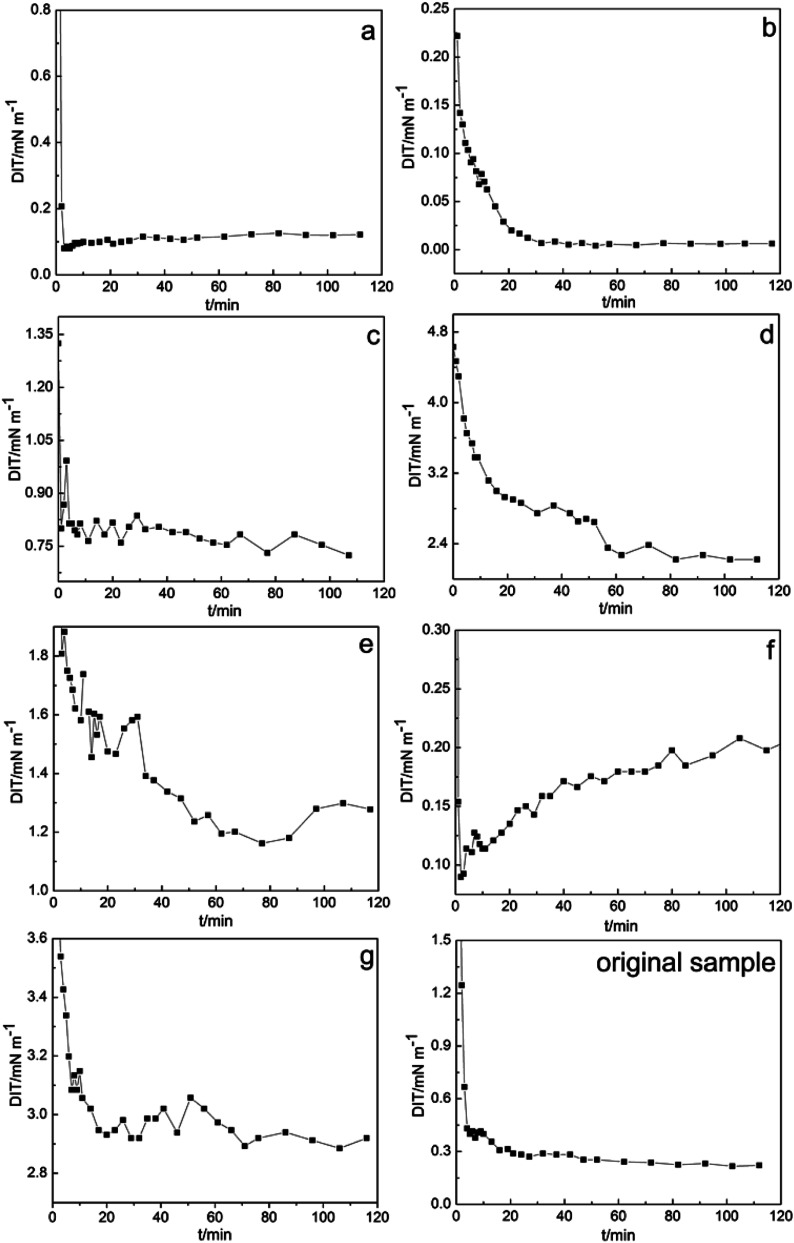
DIF results of components a–g.

In chemical flooding, DIT is a critical parameter for evaluating the oil displacement performance of petroleum sulfonate. Fig. 2 shows the DIT results of the high and low polarity sulfonate components of Daqing oilfield crude oil at different times. The calculated DIT of the original sample and components a–g were 0.226, 0.122, 5.983 × 10^−3^, 0.755, 2.264, 1.239, 0.207 and 2.915 mN m^−1^, respectively.

As can be seen from the DIT results, compared with the high polarity the components, low polarity components exhibit high capacity and efficiency for lowering the interfacial tension. In the low polarity components, the DIT of component b was reduced to an ultra-low value (10^−3^ mN m^−1^). In contrast, component g has the worst ability for lowering the interfacial tension. It can be speculated that the different polarities in the composition of petroleum sulfonate actives are an important factor affecting the interfacial activity of petroleum sulfonate (Table 3).

**Table 3:** Column chromatography results of high polarity components

Component number	Eluent	Mass fraction/%
e	Ethyl acetate	12.48
f	Isopropanol	12.06
g	Deionized water	14.09

### 3.3 The emulsification stability (ES) of components a–g

For application at the reservoir temperature (between 50 °C to 120 °C), the emulsification stability was determined at the temperature of 90 °C. Table 4 shows the emulsification stability (ES) of the separated samples at the temperature of 90 °C, where component b, which showed an ultra-low interfacial tension, has appropriate emulsifiability.

**Table 4:** The emulsifying performance of components a–g

No.	*V*_w1_ (mL)	*V*_2_ (mL)	*S*_w1_ %	*S*_te_ %
a	3.7	5.0	74	26
b	3.6	5.0	72	28
c	3.9	5.0	78	22
d	3.9	5.0	78	22
e	4.0	5.0	80	20
f	3.6	5.0	70	28
g	4.1	5.0	82	18

### 3.4 Thermogravimetric analysis (TGA) and differential thermal analysis (DTA) of petroleum sulfonate components

The thermal stability of the petroleum sulfonates components was measured using a Mettler Toledo 851° model thermogravimetric/synchronous differential thermal analyzer. The analyzer determines the weight loss of the petroleum sulfonates components at different temperatures. The experiment was conducted in the temperature range of 25 °C to 600 °C at a heating rate of 10 °C min^−1^.

**Fig. 3:**
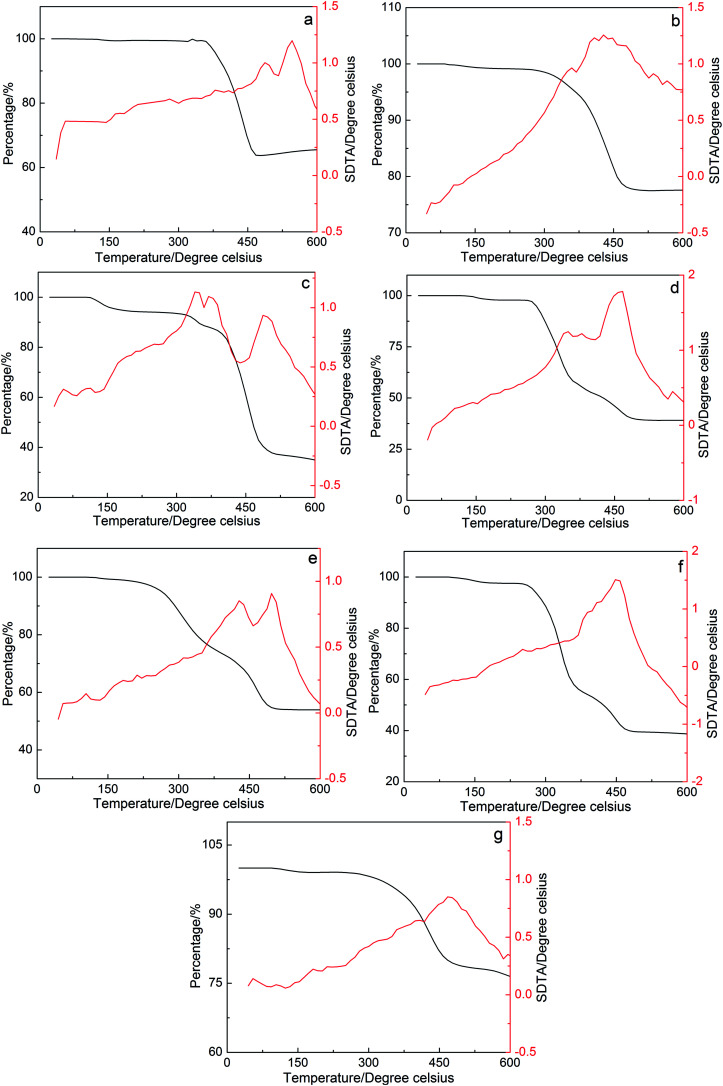
Thermal stability curves of components a–g.

The TGA and DTA analysis of the petroleum sulfonates components is illustrated in Fig. 3, where the percentage of weight loss change with an increase in temperature. The result for component b, which exhibits an ultra-low interfacial tension, shows that the first thermal loss occurred from 100 °C°C to 200 °C. There was about a 1.01% weight loss and an exothermic peak on the DTA curve of component b, which correspond to the decomposition of water molecules and residual organic solvents. Then, a 21.76% thermal loss and an apparent exothermic peak on the DTA curve of component b was observed in the second region from 300 °C to 450 °C, which can be attributed to the degradation of some light components in petroleum sulfonates at that temperature. There was an average of 77.23% ingredients of component b that was thermally stable up to 600 °C. This result shows that the components of petroleum sulfonates have good thermal stability even at a relatively high temperature. In practical application, the petroleum sulfonates components are very stable at the reservoir temperature (between 50 °C to 120 °C).^22,23^ Therefore, the petroleum sulfonates components are thermally stable at the desired reservoir temperature for enhanced oil recovery.

### 3.5 Infrared spectra of petroleum sulfonate components

**Fig. 4:**
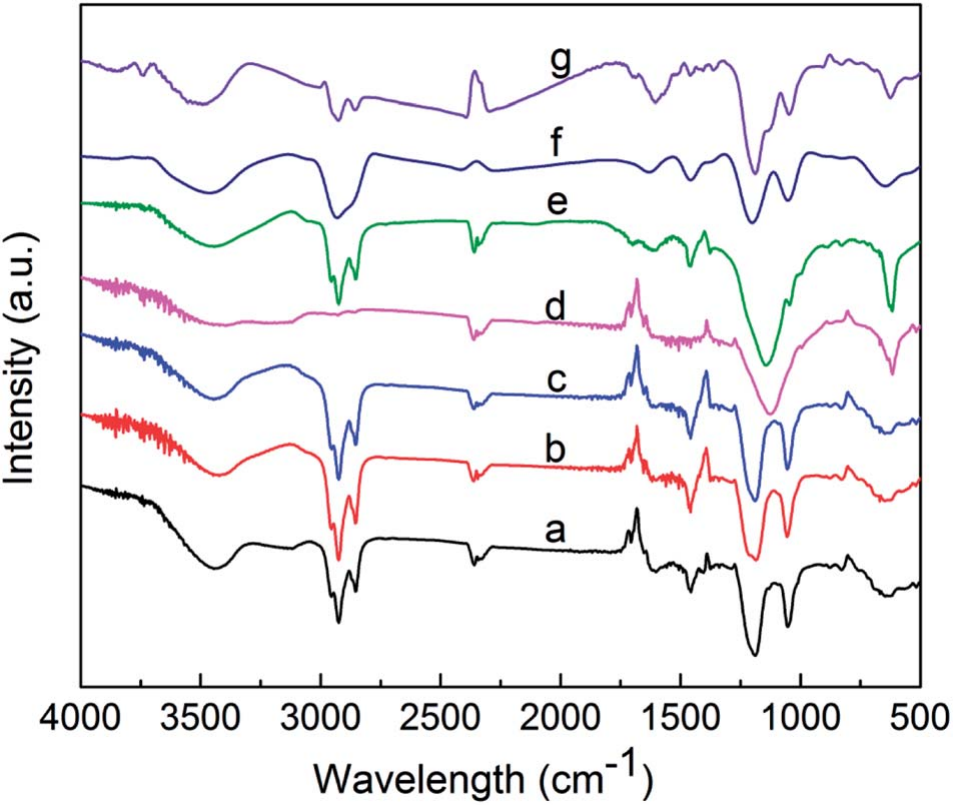
Infrared spectra of components a–g.

To determine the structural properties of the components, their infrared spectra were obtained using a Fourier transform infrared (FTIR) spectrophotometer (Nicolet iS10, Thermo, USA). Fig. 4 shows peaks at around 1051 and 1197 cm^−1^, which correspond to the symmetric and asymmetric stretching vibrations of the S=O bond of sulfonate, respectively.^24^ The peaks at 1460 cm^−1^ are attributed to the asymmetric deformation vibration and symmetric deformation vibration of –CH_3_.^25^ The peaks close to 1631 cm ^−1^ are due to the stretching vibration of the C=C bond.^26^ The asymmetric stretching vibrations of –CH_2_– and –CH_3_ appeared at 2856 and 2929 cm^−1^, respectively.^27^ The peak close to 3460 cm^−1^ for each component may be caused by traces of water in the samples.^28^ The results indicate that the infrared spectrum of each component exhibits characteristic peaks at 1051 and 1197 cm^−1^, which correspond to the sulfonic acid groups.

### 3.6 Relative molecular mass and distribution of separated components

**Fig. 5:**
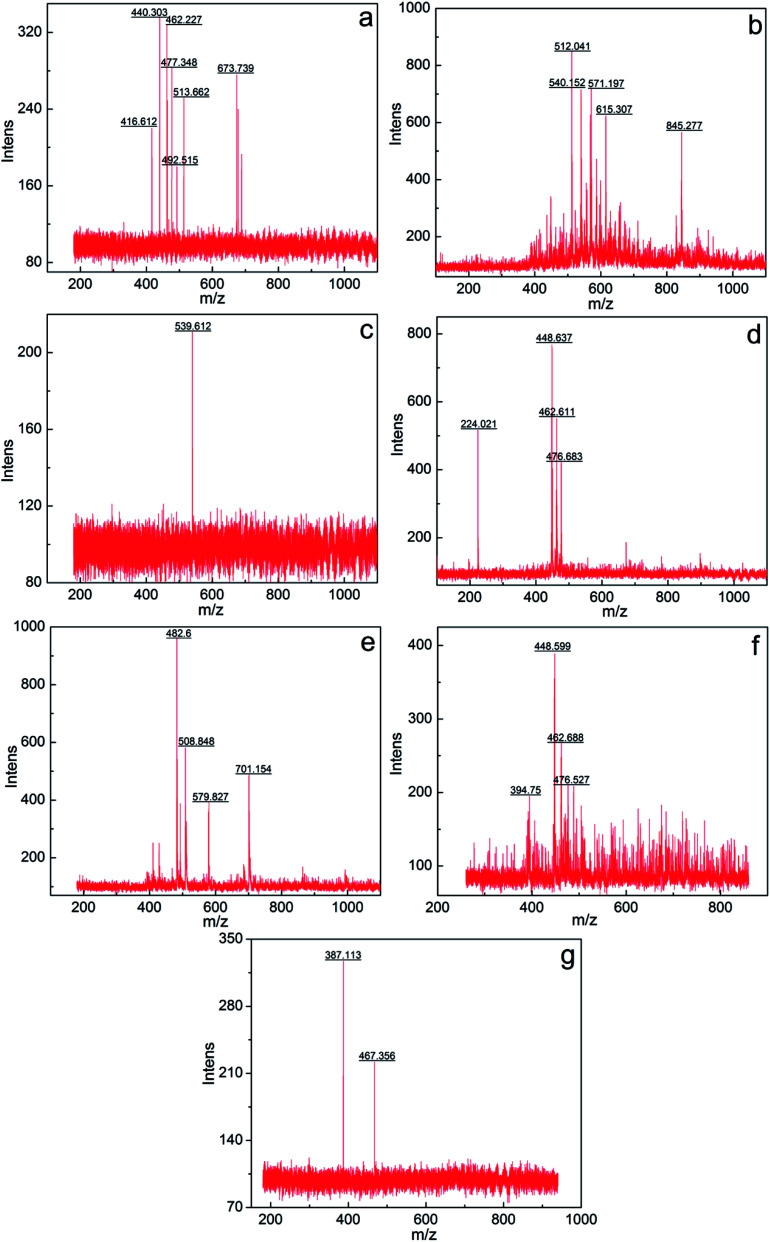
Mass spectrogram of components a–g.

The components a–g were further analyzed *via* mass spectrometry (Bruker BIFL EX III, Bruker, Germany). Fig. 5 intuitively shows the relative molecular mass distribution of components a–g, where their relative molecular masses are mainly distributed between the mass-to-charge ratio of 300 and 600. The average relative molecular mass of components a–g was calculated using eqn (3):3
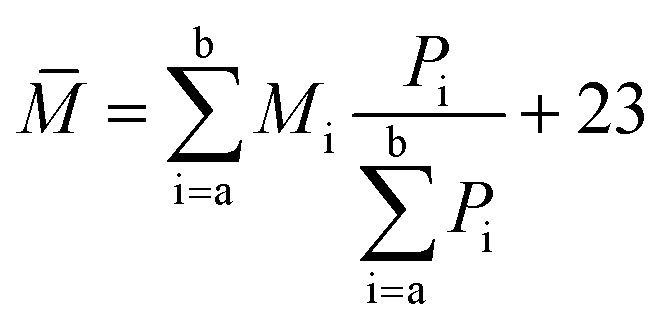
*M*_i_ – mass to charge ratio of a certain particle; and *P*_i_ – abundance corresponds to *M*_i_.

The calculated average relative molecular mass of components a–g are 519, 626, 562, 429, 571, 471 and 443, respectively.

### 3.7 Structural analysis of alkyl radical and aromatic hydrocarbon

Components a–g were characterized *via* nuclear magnetic resonance (NMR) (Bruker DPX 400, Bruker, Germany). Due to the differences in the raw oil and the process of sulfonation, the structure and compositions of the actual products are different and complex. Thus, in this study the structural parameters of average carbon number (*C̄*), branching degree of alkyl chain (BI), aromatic-carbon ratio (*f*_A_), average aromatic carbon number (*C̄*_A_), average saturated carbon number (*C̄*_S_) and average methyl number (*N̄*_CH_3__) were used to research the relationship between the structure and properties of petroleum sulfonate.

(1) Average carbon number (*C̄*):

Assuming that all the carbons are aromatic carbons, the superior limit of the average carbon number was calculated using eqn (4):4
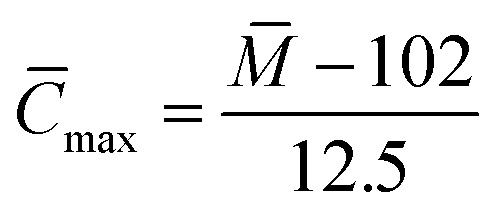


The superior limit of the average carbon number of components a–g was calculated to be 33.36, 41.92, 36.8, 26.16, 37.52, 29.52 and 27.28, respectively.

If it is assumed that all the carbons are aliphatic carbons, the inferior limit of the average carbon number can calculated using eqn (5):5
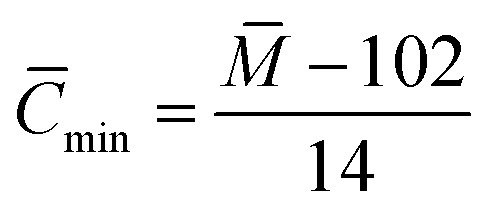


The calculated inferior limit of the average carbon number of components a–g is 29.79, 37.43, 32.86, 23.36, 33.5, 26.36 and 24.36, respectively.

**Fig. 6:**
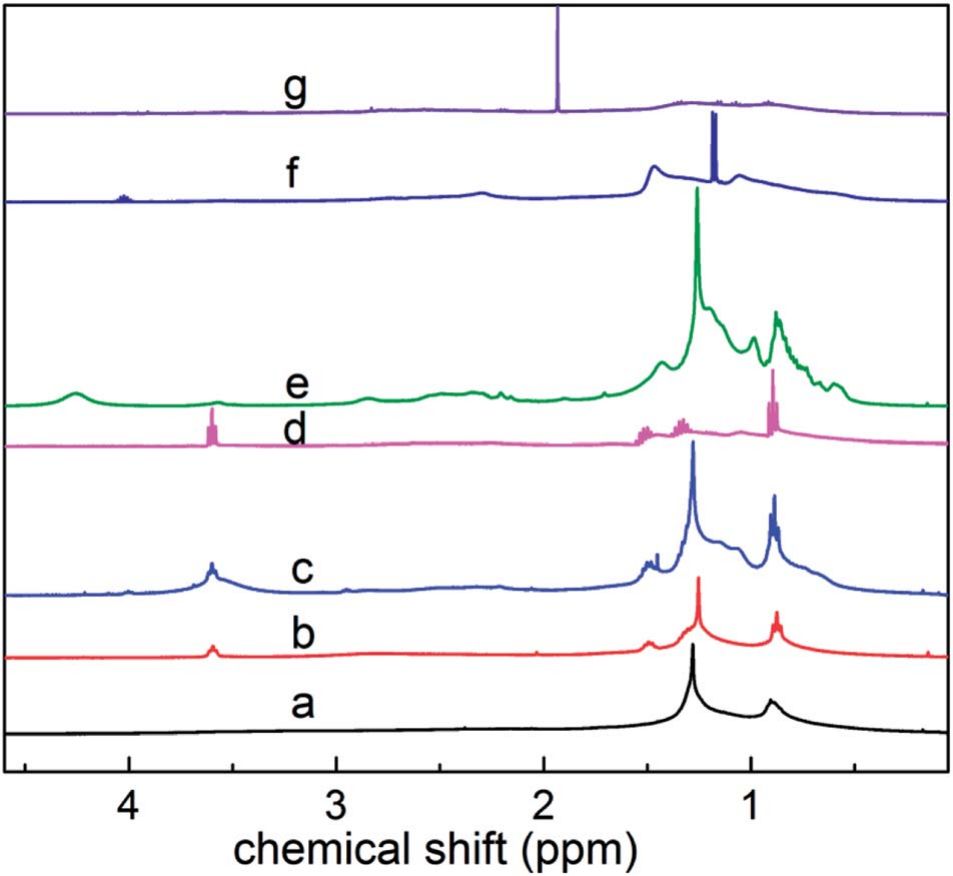
^1^H NMR spectra of components a–g.

(2) Branching degree of alkyl chain (BI):

As shown in Fig. 6, based on the data obtained from the ^1^HNMR spectra, the branching degree was calculated using eqn (6):6
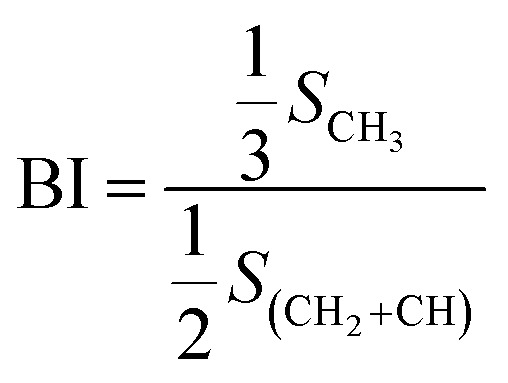
where, *S*_CH_3__ – peak area where the chemical shift ranges between 0.5 and 1.0, indicating the fraction of methyl hydrogen; *S*_(CH_2_+CH)_ – peak area where the chemical shift ranges between 1.0 and 3.5, indicating the fraction of methylene and methyne hydrogen.

The calculated branching degrees of components a–g are 0.207, 0.213, 0.227 0.293, 0.220, 0.180 and 0.160, respectively. The results show that BI is a factor that can influence the polarity. From the results, the branching degree of the low polarity components is basically higher than that of the high polarity components.

**Fig. 7:**
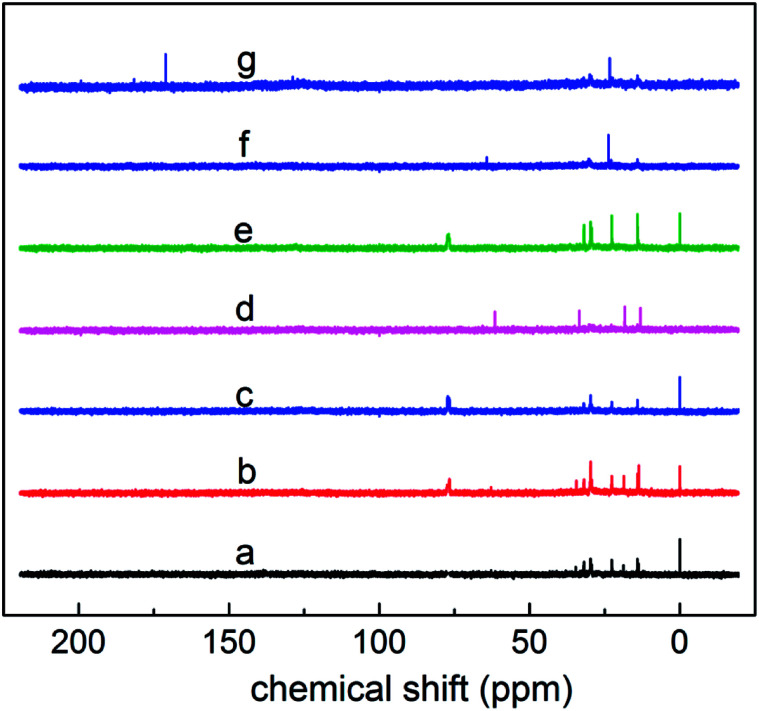
^13^C NMR spectra of components a–g.

(3) Aromatic-carbon ratio (*f*_A_):

The proportion of carbons on the aromatic ring of the alkyl aromatics in the total carbon was characterized using the aromatic-carbon ratio. According to the data obtained from Fig. 7 (^13^C NMR spectra), it was calculated using eqn (7):7
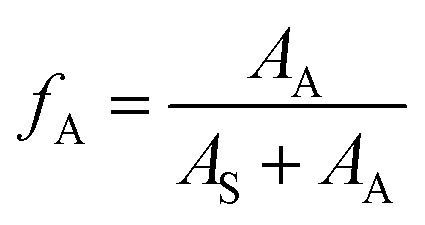
where, *A*_A_ – peak area in the ^13^C NMR spectrum, where the chemical shift ranges between 110 and 150, indicating the area of aromatic-carbon; and *A*_S_ – peak area in the ^13^C NMR spectrum, where the chemical shift ranges between 0 and 50, indicating the area of saturated carbon.

The calculated aromatic-carbon ratio of components a–g was 0.345, 0.027, 0.408, 0.296, 0.162, 0.272 and 0.102, respectively.

(4) Average aromatic carbon number (*C̄*_A_):

8 (*C̄*_A_) = (*C̄*) × (*f*_A_)

(5) Average saturated carbon number (*C̄*_S_):

9 (*C̄*_S_) = (*C̄*) − (*C̄*_A_)

(6) Average methyl number (*N̄*_CH_3__): 10
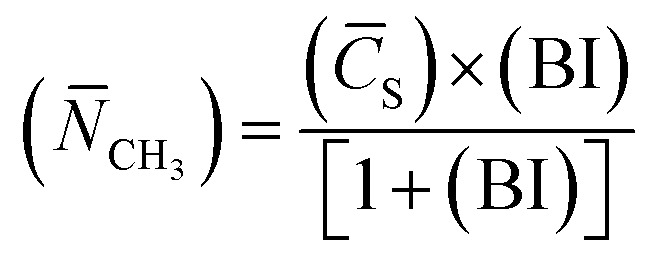


In summary, the component b achieved an ultra-low interfacial tension; thus, it can be speculated that petroleum sulfonate component b plays an important role in the oil displacement process. According to the results, the average relative molecular mass of components b is 626, and its average saturated carbon number is 36.42–40.79, indicating that its alkyl side chain contains an average of 36–40 carbon atoms. Additionally, its average methyl number is 6.40–7.16, and its alkyl chain contains an average of 6 branched chains.

**Fig. 8:**
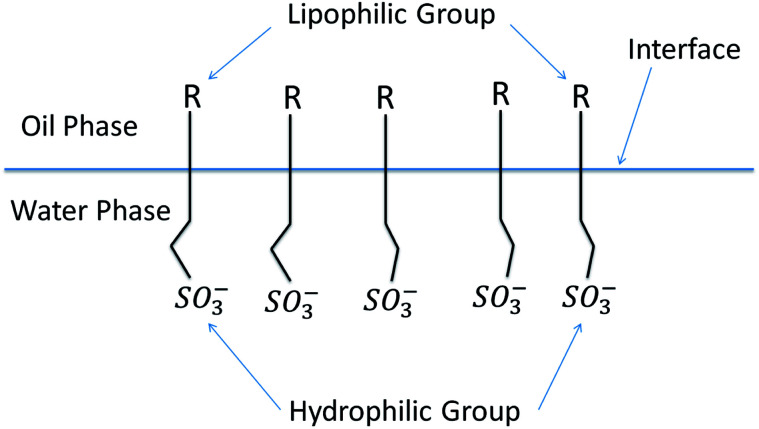
Schematic diagram of the interaction of petroleum sulfonates with oil and water.

As shown in Fig. 8, surfactant molecules consist of two distinct parts: a polar part responsible for hydrophilicity and an oleophilic nonpolar part. This special structure causes surfactant molecules to be easily adsorbed on the oil–water interface in solution to form a monomolecular film with an oriented arrangement structure. Due to the oriented adsorption of surfactant molecules on the oil–water interface, surfactants have unique surface activity for lowering the interfacial tension between oil and water.^29^

The interaction between the surfactant hydrophobic groups is the driving force for the formation of micelles, and the hydrophobic interaction between surfactant molecules increases as the length of the hydrophobic chain increases. Consequently, the oil solubility is enhanced, the surfactant molecules are more easily adsorbed on the interface and the critical micelle concentration (CMC) is reduced. Component b has the highest average saturated carbon number (*C̄*_S_) among the components. Thus, the interaction between its hydrophobic groups was enhanced, and its capacity for lowering interfacial tension was improved. Meanwhile, the arrangement and compactness of the hydrophobic chains in the interfacial adsorption layer have a great influence on the interfacial tension. As shown in Table 5, component b the highest average methyl number (*N̄*_CH_3__) among the components. Therefore, the coverage rate of hydrophobic groups (such as –CH_3_) on the oil–water interface was higher and the C–H chain is arranged more compactly. Thus, the interfacial tension was reduced.

**Table 5:** The structural parameter results of components a–g

No.	Average carbon number (*C̄*)	Branching degree (BI)	Aromatic-carbon ratio (*f*_A_)	Average aromatic carbon number (*C̄*_A_)	Average saturated carbon number (*C̄*_S_)	Average methyl number (*N̄*_CH_3__)
a	29.79–33.36	0.207	0.345	10.28–11.51	19.51–21.85	3.35–3.75
b	37.43–41.92	0.213	0.027	1.01–1.13	36.42–40.79	6.40–7.16
c	32.86–36.80	0.227	0.408	13.23–15.01	19.63–21.79	3.63–4.03
d	23.36–26.16	0.293	0.296	6.91–7.74	16.45–18.42	3.73–4.17
e	33.50–37.52	0.220	0.162	5.43–6.08	28.07–31.44	5.06–5.67
f	26.36–29.52	0.180	0.272	7.17–8.03	19.19–21.49	2.93–3.28
g	24.36–27.28	0.160	0.102	2.48–2.78	21.88–24.50	3.02–3.38

## 4 Conclusion

In conclusion, seven components of petroleum sulfonate actives were successfully subdivided *via* column chromatography. The experimental results show that component b has an ultra-low interfacial tension value (5.983 × 10^−3^ mN m^−1^) and appropriate emulsifiability. Also, the structural parameters of component b revealed its average relative molecular mass ranges from 560–626, its average alkyl side chain contains 36–40 carbon atoms and its alkyl chain contains an average of 6 branched chains, which is a suitable structure for enhancing oil recovery performance. Furthermore, the petroleum sulfonate components showed good thermal stability at the reservoir temperature. This study successfully demonstrates a reliable evaluation method for petroleum sulfonate and provides scientific information for the production of effective component in petroleum sulfonates.

## Conflicts of interest

There are no conflicts to declare.

## Supplementary Material

## References

[cit1] He L., Lin F., Li X., Sui H., Xu Z. (2015). Chem. Soc. Rev..

[cit2] Dong W., Sun D., Li Y., Wu T. (2018). Environ. Sci.: Water Res. Technol..

[cit3] Zhang D., Zhang P. Y., Zou H. K., Chu G. W., Wu W., Zhu Z. W., Chen J. F. (2010). Chem. Eng. Process..

[cit4] Weng Z., Zhang P. Y., Chu G. W., Wang W., Yun J., Chen J. F. (2015). Can. J. Chem. Eng..

[cit5] Zhan W., Zhang P., Chu G., Zou H., Yun J., Chen J. (2015). China Pet. Process. Petrochem. Technol..

[cit6] Sandvik E. I., Gale W. W., Denekas M. O. (1977). Soc. Pet. Eng. J..

[cit7] Zhao Z., Bi C., Li Z., Qiao W., Cheng L. (2006). Colloids Surf., A.

[cit8] Tsubouchi M., Yamasaki N., Yanagisawa K. (1985). Anal. Chem..

[cit9] Li Z. P., Gong X. M., Li Q. Y. (1984). Chin. J. Anal. Chem..

[cit10] Olajire A. A. (2014). Energy.

[cit11] Hou Z., Li Z., Wang H. (2000). Colloids Surf., A.

[cit12] Zhu Y. W., Zhao R. H., Jin Z. Q., Zhang L., Zhang L., Luo L., Zhao S. (2013). Energy Fuels.

[cit13] Zhou H., Luo Q., Gong Q. T., Liu Z. Y., Liu M., Zhang L., Zhao S. (2017). Colloids Surf., A.

[cit14] Yu F., Fan W. Y., Nan G. Z., Li S. P., Duan Y. Z. (2008). Acta Pet. Sin..

[cit15] Márquez N., Gonzalez S., Subero N., Bravo B., Chavez G., Bauza R., Ysambertt F. (1998). Analyst.

[cit16] Padula L., Balestrin L. B. D. S., Rocha N. D. O., de Carvalho C. H. M. (2016). Energy Fuels.

[cit17] Chan K. S., Shah D. O. (1980). J. Dispersion Sci. Technol..

[cit18] Jia Z., Yuan W., Zhao H., Hu H., Baker G. L. (2014). RSC Adv..

[cit19] Li W. T., Nan W. H., Luo Q. L. (2014). RSC Adv..

[cit20] Zhao Z., Li Z., Qiao W., Cheng L. (2005). Colloids Surf., A.

[cit21] Zhang Q. Q., Cai B. X., Gang H. Z., Yang S. Z., Mu B. Z. (2014). RSC Adv..

[cit22] Babu K., Maurya N. K., Mandal A., Saxena V. K. (2015). Braz. J. Chem. Eng..

[cit23] Saxena N., Pal N., Ojha K., Dey S., Mandal A. (2018). RSC Adv..

[cit24] Deimede V., Voyiatzis G. A., Kallitsis J. K., Qingfeng L., Bjerrum N. J. (2000). Macromolecules.

[cit25] Li X., Zhu Z., Zhao Q., Wang L. (2011). J. Hazard. Mater..

[cit26] Zhu L., Lu Y., Wang Y., Zhang L., Wang W. (2012). Appl. Surf. Sci..

[cit27] Arunagirinathan M. A., Roy M., Dua A. K., Manohar C., Bellare J. R. (2004). Langmuir.

[cit28] Fang M., Wang K., Lu H., Yang Y., Nutt S. (2009). J. Mater. Chem..

[cit29] Samanta A., Ojha K., Sarkar A., Mandal A. (2011). J. Pet. Eng. Technol..

